# VISION IMPAIRMENT IN A GLOBAL LIFE COURSE MODEL OF POTENTIALLY MODIFIABLE DEMENTIA RISK FACTORS

**DOI:** 10.1093/geroni/igac059.1498

**Published:** 2022-12-20

**Authors:** Joshua Ehrlich, Jenna Goldstein, Bonnielin Swenor, Heather Whitson, Kenneth Langa, Phillip Veliz

**Affiliations:** University of Michigan, Ann Arbor, Michigan, United States; University of Michigan, Ann Arbor, Michigan, United States; Johns Hopkins University, Baltimore, Maryland, United States; Duke University, Durham, North Carolina, United States; University of Michigan, Ann Arbor, Michigan, United States; University of Michigan, Ann Arbor, Michigan, United States

## Abstract

Vision impairment (VI) is a risk factor for accelerated cognitive decline and incident dementia. An estimated 90% of VI is preventable. Nonetheless, VI has not been included in the dominant life-course models of dementia risk factors. We sought to strengthen existing models of potentially modifiable dementia risk factors through the inclusion of VI using cross-sectional survey data from the Gateway to Global Aging project (G2Aging) from 31 countries. Prevalence rates and communalities from the G2Aging will be used to estimate the population attributable fraction (PAF) of dementia due to VI. It is expected that the PAF for VI will range from 1.0%-2.0%, suggesting that >10,000,000 prevalent dementia cases globally may potentially have been prevented through healthy vision. Since a large majority of VI can be treated with cost-effective but underutilized interventions, this may represent a viable target for future interventional research that aims to slow cognitive decline and prevent dementia.

